# Midlife Transition Experiences of South Asian Immigrant Women in Canada: A Qualitative Exploration

**DOI:** 10.1177/08445621231153525

**Published:** 2023-02-12

**Authors:** Ping Zou, Arzoo Alam, Jing Shao, Yan Luo, Yanjin Huang, Hui Zhang, Wei Wang, Souraya Sidani

**Affiliations:** 1 School of Nursing, 6057 Nipissing University, Toronto, Ontario, Canada; 2 Dalla Lana School of Public Health, 7938 University of Toronto, Toronto, ON, Canada; 3 Institute of Nursing Research, Zhejiang University School of Medicine, Hangzhou, Zhejiang, China; 4 Faculty of Nursing, Health Science Center, Xi’an Jiaotong University, Xi’an, Shaanxi, P. R. China; 5 School of Nursing, 34706 University of South China, Hengyang, Hunan, P. R. China; 6 Department of Cardiology, Guizhou provincial people's Hospital, Guiyang, Guizhou, China; 7 Toronto Western Hospital Bariatric Surgical Program, University of Health Network, Toronto, Canada; 8 Lawrence S. Bloomberg Faculty of Nursing, 7938 University of Toronto, Toronto, ON, Canada; 9 Daphne Cockwell School of Nursing, 7984 Ryerson University, Toronto, Ontario, Canada

**Keywords:** Midlife, transition, women, immigrant, qualitative, South Asian, Canada

## Abstract

**Background:**

South Asians make up a significant portion of the immigrant population in Canada, and a large portion of them are in their midlife. To improve the midlife transition of South Asian immigrant women, it is necessary to understand their lived experiences.

**Purpose:**

Guided by the transition theory, this study investigates the midlife experiences of South Asian immigrant women in Canada.

**Methods:**

Twenty-two South Asian midlife, immigrant women were recruited to participate in this study from the Greater Toronto Area, Ontario, Canada. This study consisted of one asynchronous online focus group with 12 participants and ten one-on-one telephone interviews. Qualitative content analysis was guided by transition theory.

**Results:**

South Asian immigrant women experienced many different transitions in their midlife in Canada. These transitions included changes in their (a) lifestyle, (b) career, (c) family, (d) physical health, (e) mental health, (f) social, (g) environment, and (h) personal development. Women actively managed their transitions using strategies such as exercise, socialization, counseling, and religion. Women expressed the need for social, community, and governmental support to facilitate their midlife transitions.

**Conclusion:**

To promote healthy midlife transition, governments need to create better employment policies to facilitate immigrant women settlement, transferring skills, and re-employment in Canada. In addition, health care and community services to promote physical and mental health should be emphasized.

## Introduction

The immigrant population in Canada has gradually increased over the past few decades, with 42.4% of immigrants migrating in the past 20 years. Immigrants compose 21.9% of the Canadian population (Statistics Canada, 2017b). South Asians make up a significant portion of the immigrant population, a large portion of them are in their midlife. The ethnic category of South Asian refers to those whose cultural background originates from one of the following countries: Afghanistan, India, Pakistan, Bangladesh, Sri Lanka, Nepal, Bhutan, or Maldives. The South Asian population in Canada more than tripled between 1981 and 2001 largely due to an increase in immigration(Tran et al., 2005). In 2006, the South Asian population overtook the Chinese population as the largest visible minority population(Government of Canada, 2008). As of the 2016 census, they comprise 25.1% of the visible minority population in Canada and 5.6% of the total population (Statistics Canada, 2017a). The Canadian government has also launched a recent initiative to welcome at least 40,000 refugees from Afghanistan, with 15, 715 welcomed as of June 2022(Immigration & Citizenship, 2021).

A significant health challenges women face during midlife is menopause, which typically occurs between the ages of 45 and 55 (Dalal & Agarwal, 2015). Menopause is defined as 12 months of amenorrhea in the absence of other explanations, signifying the end of menstruation and reproductive abilities (Minkin, 2019). During the menopausal transition, a decrease in estrogen levels are associated with (a) vasomotor symptoms by contributing to dysfunction of central thermoregulation(Talaulikar, 2022) (b) vaginal dryness and irritability through the thinning of vaginal mucosa (Talaulikar, 2022) (c) mental health symptoms such as anxiety and depression, potentially through central nervous system effects (Walf & Frye, 2006). Aside from hormonal changes, personal factors, including a history of depression, adverse life events, and negative attitudes towards menopause have been found to be risk factors for development of depressive symptoms in menopause (Vivian-Taylor & Hickey, 2014).

Aside from menopause, midlife transitional experiences include a variety of changes. Many women in this stage develop the feeling of “empty nest”, describing a sense of loss when children move out (Dare, 2011). Women may face multiple other life stressors, including employment problems, family issues, and social stress; all of which interact to create a stressful midlife transition (Thomas et al., 2018). Women in their midlife play multiple roles, and balancing these responsibilities has been a commonly reported challenge (Thomas et al., 2018). Middle-aged women have frequently reported that their roles revolving around family, work, and eldercare act as sources of stress (Kenney, 2000). An increasing number of women in this stage are also undergoing divorce and its associated role changes, attributable in part to the culmination of different stressors and also increased introspection leading to re-evaluation of relationships(Dare, 2011). Overall, due to the substantial psychological and physiological changes that occur during this phase of life, more attention should be placed on healthy midlife transition.

In addition, the process of immigration can increase the stress experienced by women in their midlife. They may undergo acculturative stress, which refers to the difficulties immigrants face when adapting to a new culture and lifestyle (George et al., 2015). Migration can alter the social, cultural, and political context of a woman's menopausal experience, and introduce her to different views about the midlife transition (Spitzer, 2009). The immigration experiences of South Asian women are also distinct. A number of sociocultural factors for South Asian immigrants increase risks of domestic violence in this population (Rai & Choi, 2018). Additionally, political unrest and humanitarian crises in Afghanistan greatly contribute to psychological distress due to trauma, further complicating the migration narratives of some South Asian women(Alemi et al., 2014). Migrational stressors, together with menopausal symptoms and other changes occuring during midlife, contribute to making menopause a challenging time for immigrant women.

To improve the midlife transition of South Asian immigrant women, it is necessary to understand their lived experiences. Previous studies have explored this issue; however, the evidence is inconsistent and insufficient to provide adequate direction on how to best support this population during this period of time. George (1988) examined the menopausal experiences of Sikh women in Vancouver, British Columbia, Canada. Participants in this study had positive experiences since they kept similar social and cultural lifestyles. However, this study is outdated, and the results may not provide comprehensive insight into experiences from all provinces in Canada. Another study examined the midlife experiences of South Asian women living in the United Kingdom, compared to Caucasian women living in the United Kingdom, and South Asian women living in India (Hunter et al., 2009). The study found that the social meaning of menopause was remarkably similar for the two South Asian groups, suggesting that culture is not bounded by geography or location. However, Canadian immigrant experiences may vary due to different immigration policies and newcomer resources/supports. Lastly, a recent study explored the menopausal experiences of migrant women in Australia and Canada (Ussher et al., 2019). Contrastingly, South Asian participants in this study perceived menopause as a negative phenomenon and had limited knowledge on the topic due to secrecy and taboo culture. Additionally, a scoping review on the menopausal experiences of South Asian immigrant women globally found both positive and negative experiences but did not investigate in-depth other midlife transitions besides menopause (Zou et al., 2022). It is evident that women around the world have differing views and perceptions about the midlife transition. There exists a research gap in how South Asian immigrant women living in Canada specifically view and experience their midlife transition.

## Transition theory

The development of the transition theory was initiated in the 1960s when Dr. Afaf Meleis was working on her PhD thesis and began to question the nature of transitions in human experiences. Transitions have been defined as a process humans undergo when faced with changes in their lives or environments (Schumacher & Meleis, 1994). These experiences are shaped by personal and environmental factors, including the expectations and perceptions of the individual, the meanings they attribute to the experiences, their knowledge and skills in handling the change, and their physical and mental well-being (Chick & Meleis, 1986; Schumacher & Meleis, 1994) . Based on her early work, Meleis collaborated with her colleagues to further develop the concept of transition by performing concept analysis and, thus, developed the emerging middle-range theory (Chick & Meleis, 1986; Meleis et al., 2000a).

Major concepts of transition theory include the types of transitions, properties that characterize transition experiences, conditions that determine the nature of responses, indicators of healthy responses, and nursing therapeutics. There are four types of transitions: developmental, situational, health/illness, and organizational (Chick & Meleis, 1986; Schumacher & Meleis, 1994). Transition conditions, such as personal, community, societal, and global, are circumstances that influence the way a person moves through a transition (Meleis et al., 2000a). Patterns of response include process and outcome indicators, which describe healthy responses to transitions (Meleis et al., 2000a). Nursing therapeutics are measures or interventions that facilitate healthy transitions (Schumacher & Meleis, 1994). The middle-range transition theory has been utilized to explore various health care issues and further develop situation-specific theories on (a) the migration transition for migrant farmworker women; (b) the transition to adult day health services; (c) guiding interventions for people with heart failure; (d) care transitions; (e) well-being in refugee women experiencing cultural transition; (f) pain experience for Asian American cancer patients (Baird, 2012; Bull & McShane, 2008; Clingerman, 2007; Davidson et al., 2007; Geary & Schumacher, 2012; Im et al., 2008). It is evident that transition theory is versatile and can be used to explain a multitude of transitional periods. Specifically, this theory has been used to describe the menopausal transition of Korean and Asian immigrant women in the United States (Im & Lipson, 1997).

In this study, transition theory was used as a framework to guide research question formation, data collection, data analysis, and discussion of the findings. The purpose of our study is to describe the midlife transitional experiences of South Asian immigrant women in Canada guided by transition theory. The midlife transition refers to the various health, family, and work changes that affect an individual's identity during their middle-aged years (Barrett, 2005). It is often characterized by key events such as menopause, empty nest syndrome, and having grandchildren (Lachman & James, 1997). For women in particular, the midlife transition can have negative connotations due to issues surrounding ageism, body image, and the aging process (Saucier, 2004). This study's findings provide valuable information on the diverse experiences of South Asian immigrant women in their midlife, which can help communities improve the quality of life for South Asian immigrant women living in Canada.

## Methods

This study followed a qualitative approach and consisted of one asynchronous online focus group with 12 participants (Im & Chee, 2012) and ten one-on-one telephone interviews (Gill et al., 2008). Qualitative content analysis guided by transition theory was used. The focus group aimed to gain multiple perspectives and stimulate interactive discussion. The individual interview focused on understanding personal experience and perception. The combination of focus group and individual interviews ensured that participants choose the method most convenient and appropriate for them, so that they may feel comfortable sharing their experiences during the data collection process. It also provided researchers an opportunity to fully explore the complexities of the women's menopausal experiences and of the transition conditions occurring in both group and individual settings.

This study was conducted with immigrant women from South Asian communities in the Greater Toronto Area, Ontario, in collaboration with non-profit agencies and community centers which provide immigrant settlement services. Inclusion criteria included: (a) 45 to 60 years old, (b) self-identification as a first-generation South Asian immigrant, (c) self-identification as a woman, and (d) self-identification as born outside of Canada. Temporary visitors (e.g., migrant workers, international students) were excluded. Using purposeful sampling (Polit & Beck, 2016), Twenty-two immigrant women were recruited. This sample size is appropriate to reach information saturation in qualitative studies (Creswell & Poth, 2017).

The telephone interviews and focus groups were conducted by trained researchers (AA & PZ). Menopausal experiences were explored guided by seven discussion topics, including a) women's daily life schedules and hardships; (b) cultural descriptions of menopausal symptoms; (c) women's culture-specific attitudes and responses to menopausal symptoms; (d) women's perceived culture-specific causes of menopausal symptoms and management strategies for menopausal symptoms; (e) life events influencing women's menopausal symptom experiences in their daily lives; (f) women's preferences for symptom management; (g) women's suggestions for community social services to support menopausal process. The seven discussion topics were built upon previous studies (Im & Chee, 2006; Kagawa–Singer et al., 2002) and consultation of subject experts. The focus groups were hosted in a social media platform, with which participants were familiar. Exclusive groups were created to allow a secured, confidential and safe environment for research participants (Robinson et al., 2019; Skelton et al., 2020). Each topic was posted on an online forum by the researchers in a serial fashion across the two-week period of data collection. The participants posted their comments to the questions and responded to each other as well. Each individual telephone interview lasted for 30–45 min and was taped. Each participant was offered a $15 gift card to recognize her time and effort.

The data analysis were conducted by trained researchers (AA & PZ). While the qualitative data analysis was characterized by the simultaneous collection and analysis of data (Sandelowski, 1995), data analysis of this study began after the first two interviews were completed (Patton, 2002). Data analysis was guided by transition theory. Qualitative content analysis, which offers a comprehensive data summary with minimal interpretation, was conducted (Sandelowski, 2010). Data from interviews and online focus groups were transcribed into word processing files. The data were then analyzed following the general approach of content analysis suggested by Krueger and colleagues (Krueger & Casey, 2014). The transcripts were read several times by each researcher to gain a comprehensive understanding of the collected data. Each researcher then independently performed thematic coding to identify meaningful text. Lastly, data triangulation was completed by two researchers to reach a consensus over the themes.

Ethical approval was obtained from Nipissing University Research Ethics Board. Informed consent was obtained from all study participants, who were fully informed about the purpose and process of the study. If a participant were emotionally disturbed during an interview, the researcher would stop the interview and refer the participant to related psychosocial services in the community.

## Results

### Participant characteristics

Participants’ (n = 22) characteristics are reported in Table 1. The women's mean age was 48.5 (SD = 3.4) years. Participants had been living in Canada for an average of 12 (SD = 5.8) years. Most participants were married (n = 17, 77.3%), employed full-time (n = 12, 54.5%), and earning an annual personal income between $20,001 and $50,000 (n = 10, 45.5%).

**Table 1.** Demographic characteristics of interview and focus group participants (n = 22).

Variable	Mean (SD)/Category	n	%
Age	48.5 (3.4)	N/A	N/A
Number of children	2.2 (1.0)	N/A	N/A
Length of time living in Canada (years)	12.0 (5.8)	N/A	N/A
Number of people supported by family income	3.6 (1.5)	N/A	N/A
English Proficiency (on a scale of 4-16)	15 (2.0)	N/A	N/A
Menstrual cycle - past 3 months	Regular	11	50
	Irregular	6	27.3
	No menstruation	5	22.7
Menstrual cycle - past 12 months	Regular	12	54.5
	Irregular	7	31.8
	No menstruation	3	13.6
General health	Excellent	2	9.1
	Very good	6	27.3
	Good	8	36.4
	Normal	4	18.2
	Bad	2	9.1
Health compared to 1 year ago	Much better	4	18.2
	A little better	4	18.2
	Almost the same	8	36.4
	A little worse	6	27.3
	Much worse	0	0
	Full-time	12	54.5
Employment status	Casual/Part-time	3	13.6
	Self-Employed	1	4.5
	Unemployed	5	22.7
	Not reported	1	4.5
Highest educational level	High school	0	0
	Diploma/certificate	1	4.5
	Bachelor's degree	6	27.3
	Master's degree	14	63.6
	PhD degree	1	4.5
Personal income	<$20,000	3	13.6
	$20,001 to $50,000	10	45.5
	$50,001+	7	31.8
	Not reported	2	9.1
Family income	<$20,000	1	4.5
	$20,001 to $50,000	4	18.2
	$50,001+	16	72.7
	Not reported	1	4.5
Paying for basic living expenses	Very difficult	2	9.1
	Somewhat difficult	10	45.5
	Not difficult	10	45.5
Marital status	Married	17	77.3
	Widowed	1	4.5
	Separated	2	9.1
	Other (Divorced)	2	9.1
Religious beliefs	Catholic	1	4.5
	Muslim/Islamic	15	68.2
	Other	6	27.3
Importance of religion in daily life	Extremely important	12	54.5
	Very important	7	31.8
	Somewhat important	3	13.6
Current living status	Living in own house/apartment	17	77.3
	Rent	5	22.7

### Lifestyle transition

As newcomers to Canada, the main challenges expressed by participants were restarting their lives with no help (011805-12, 011805-15, 011805-21, 080101-21) and managing a busy schedule (011805-25, 011805-12). As one participant expressed, “If I were living back home in my own country, I would’ve had a lot of help in the way of housework, but here in Canada, you have to be very self-sufficient. From the day-to-day chores, […] it was like a cocoon I was in, and when I came here, we were thrown open to a lot of things which were never done before” (011805-12). Another participant summarized this experience, “Then suddenly, to come to this country and start doing everything on your own (011805-12)”.

Women self-managed these difficulties by organizing and delegating household chores to family members and making time for themselves (011805-10, 011805-15, 10726366640). One participant explained, “I try to organize better by assigning tasks beforehand to give enough notice to kids to work things around their schedules (10726366640)”. Participants also utilized their community resources. One participant described how a French language program was helpful for her, “I was really happy to be there because they taught us a lot; they taught us how to cope with things in Canada (011805-12)”.

Future recommendations for improving the lifestyle transition include increasing the accessibility of language services. One participant suggested, “Places should offer to help [immigrants] learn languages and express themselves. […] Going door to door, if you know who the immigrants are […] invite them to participate or join those services, to understand how they should be living their life here […] The reason why they’ve come here, why they’ve chosen this place, is to have a better life, right? So, how can they make this better? It's important to know how and why (080101-21)”. Furthermore, participants also suggested that menopausal women receive care packages, "Every woman goes through it [menopause] differently, so they can order a care package of whatever their symptoms are; there could be a small booklet in it, an audio or a podcast, a little USB with a podcast they can hear (011805-18)”.

### Career transition

Despite having the necessary credentials, women often remained unemployed and had difficulty finding a suitable job since their credentials ‘from back home’ were not recognized or valued in Canada (011805-21, 011805-12, 190802-03, 10726366640, 091906-05, 10860561533). Frequently, women had to retrain themselves in a profession or had to accept a general labor position. One participant explained, “As I moved to Canada over 10 years ago, I am settled in my professional life. But it took me a long time as I did many courses to reach where I am today (091906-05)”. Other participants expressed similar sentiments, “Change of career and/or starting at an entry level position is not easy to accept mentally as well as at times physically (10726366640)”.

To cope with these challenges, many women either changed their profession (011805-24, 10726366640) or upgraded their education/skills to work in a related field (190802-03, 091906-05). Additionally, participants used job search programs offered in their communities, but did not find success because they were either overqualified or did not have any prior experience in Canada (011805-12, 190802-03). One participant shared, “Initially when we came to Canada, we used to go to the library for some job finding courses. […] They would like to offer jobs which are much lower in your caliber (190802-03)”. Lastly, due to difficulties with searching for jobs, some spouses were forced to separate to financially support their families. One participant shared, “I did not have a job for two years, and my husband had to go back to Bahrain where he was working so that at least one of us is earning. You cannot survive without a job here in Canada (190802-03)”.

Future recommendations include having more job opportunities and awareness about employment services (011805-12, 011805-18, 10857001590). One participant expressed, “I realize that, if we didn’t have the Canadian education, getting a job was very difficult. […] There are loads of job banks and stuff like that. […] But I’m thinking that if there was a place where we could do it without making a big deal out of Canadian experience or education (011805-12)”. Furthermore, many participants wished for more support and understanding from their employers (011805-21, 10860561533). One participant suggested “that after a certain age, they should increase the sick leave for people” (011805-21).

### Family transition

Many participants did not receive family support (011805-01, 011805-21, 080101-21, 10943630204, 10860561533, 10857001590, 10862149085, 011805-15, 011805-27, 10726366640). Regarding spousal support, one participant shared, “You know how you’re expecting too much from somebody, it's that kind of thing with my husband. […] At the time […] I felt he really didn’t understand as much (080101-21)”. In terms of parental support, one participant explained, “I would have discussed [my issues] with my mom, I didn’t have her to discuss with (011805-21)”. Lastly, some women found it challenging to raise their children in a new country. One participant said that it was difficult to get “words through” to her children because she feared “strong reactions [from] the younger generation, in terms of their responses (080101-21)”.

Participants identified family support as a positive influence in this transition, particularly from their spouse (011805-34, 10860561533, 011805-25, 091906-05), children (011805-24, 011805-12, 080101-21, 10726366640, 011805-25, 091906-05), parents (011805-15), aunts (011805-15, 011805-12) and cousins (011805-12). As described by one participant, “[My family and I] unitedly strive to achieve goals together. We have a family group created on WhatsApp to be in the loop at all times. We enjoy our family time together on weekends and we strive to ‘simplify’ our lives as much as possible (011805-10)”.

In terms of future recommendations, participants suggested self-care and familial support (011805-15, 10943630204). As one participant described it, “Women need to feel appreciated and loved. Kids and spouses need to value our presence more every passing day. Mother's Day drama is meaningless if other days display no positive emotions and care (011805-10).” Another participant added, “What programs can be introduced by the community or government to educate the middle-aged men? […] Most of the time middle-aged wives educating the husbands does not work. I have noticed young, married couples are more understanding and practical” (011805-01).

### Physical health transition

Participants reported general changes to their physical health (080101-21, 011805-27, 10943630204, 011805-12, 091906-05, 190802-03, 10862149085), including fatigue, decreased energy levels (10726366640, 011805-15, 011805-21, 011805-24, 011805-34), weight gain (011805-24, 011805-27, 011805-18, 10726366640, 091906-05, 011805-25, 10943630204, 10857001590, 10862149085), and hot flashes (011805-24, 011805-27). As one participant explained, “I’m aging. […] My body isn’t as active and agile as it used to be, so I feel those changes a lot… I do admire myself sometimes for the good things I do, but I really do get agitated with the fact that it's not going to go back, it's just different. You have to age (080101-21)”.

Physical health transitions were self-managed through walking (011805-15, 10726366640, 10860561533, 10862149085), swimming (10860561533), and yoga/meditation/tai chi (011805-12, 011805-34, 10862149085). One participant described, “With exercise, my body also feels lighter if it's heavy. […] I sweat and that feels good, I feel comfortable (011805-18)”. Participants also made changes to their diet by incorporating healthier food choices and reducing their junk food intake (011805-18, 10726366640, 011805-10, 091906-05).

Proposed strategies to ease this transition included social media campaigns, health promotions, and subsidized healthcare services. One individual stated, “I think media can play an important role by creating awareness not only in women on how to prepare ahead for middle age, [but] […] programs should aim to disseminate knowledge in other family and social networks to support women (10726366640)”. Furthermore, participants believed that doctors should play a role in promoting awareness and education for women experiencing this transition (10943630204, 011805-15). One participant proposed, “In my opinion, we need some substantial support from our government to help us all in the mindful aging process (10862149085)”. Participants also suggested increased physical activity, both individually and through community exercise campaigns (091906-05, 011805-10, 10857001590, 10726366640). One participant suggested, “Too idealistic of a thought probably but ‘FIT at 50’ should be launched as a campaign (10726366640)”.

### Mental health transition

Participants acknowledged that they felt more emotional during their midlife transition. One participant stated, “Sometimes, [I feel] emotional, or sometimes, depressed (011805-18)”. Similarly, another participant reflected, “There's so much change. I’m very sensitive now, I cry very easily now. (011805-24)”. One participant described her severe mood swings, “There were moments when I was immensely angry […] and then, like in the next moment when my anger was done, I would just go to a corner and cry (011805-27)”. Participants reported that they became more sensitive (011805-15, 011805-12), experienced sudden changes in their mood (011805-15), felt irritation and displeasure (011805-15, 011805-27), depressed (011805-21, 011805-18, 080101-21), or anxious (011805-15, 011805-24, 011805-12, 190802-03).

Participants coped with their mental health transitions through spiritual and religious practices (011805-15, 011805-24, 011805-18, 011805-12, 080101-21, 10726366640, 011805-10, 011805-34, 10857001590), relaxation, maintaining a positive mindset (011805-34, 10857001590, 10862149085), and counselling (080101-21, 10860561533). One participant expressed, “My relationship with God, it helps me a lot. There are certain things that I cannot, and will not, speak about with my family, with my friends, to my partner, to my children, but I can always speak to Him (011805-15).”

Participants also found relaxing activities such as sleeping (011805-21), exercising (190802-03, 011805-10), meditating (011805-10, 011805-34), watching television (190802-03), and spending time with family (10862149085) to be helpful in de-stressing. In terms of keeping a positive attitude, a participant shared, “I believe if we keep our mind and our thoughts positive and realize that it is a natural process which will take its own time to settle down then we can handle the symptoms. […] It is in our mind and mind is ours and we can control it. So, we have to be brave to not let menopause impact us (011805-01)”. To improve the mental health symptoms that arise from this transition, one of the main recommendations was for participants to explore counselling services (080101-21, 10857001590).

### Social transition

Regarding their social life transition, participants expressed that they either do not have anyone to socialize with or simply have no desire to do so (011805-15, 011805-21, 011805-24). As one participant shared, “The community I lived in Singapore, it's a very close-knit community. […] I strongly believe I would have gotten much better support [there] than Canada (011805-21)”. Another participant described, “Before, I was a very outgoing person. […] Now I want to come home most of the time, I don’t like to go out most of the time (011805-24)”. Moreover, participants were unaware of the types of community social services offered to them. As one participant described, “Well, there are none to less support or education over this issue. We need awareness in public that this is real” (10943630204).

Participants coped with their social transition by keeping in touch with their friends (011805-01, 011805-21, 190802-03, 080101-21, 10726366640, 091906-05, 011805-34, 10857001590) or attending community social events (011805-18). One participant explained, “I feel that we all know so many people all around. […] Always have one friend who can hear you from heart, it is a blessing to have such friend (011805-01)”. Another participant expressed that attending events in a public community space helped to improve happiness (011805-18).

Future recommendations included implementing community events where South Asian immigrant women can meet likeminded individuals experiencing similar challenges (011805-10, 011805-24, 011805-27, 080101-21, 091906-05, 10857001590). One participant suggested, “I think we could/should have community events/social gatherings or a forum to educate and support middle-aged women on issues like menopause. […] I think women should mix around with like-minded women and they should form a small group that meets regularly and supports one another (011805-10).” Many participants wish there was less stigma around menopause (011805-25, 091906-05) and more services to help with the transition (091906-05).

### Environmental transition

Many participants had difficulty transitioning to Canadian weather and reported that the harsh winters were inconvenient and difficult to manage (011805-24, 011805-15, 011805-12, 080101-21, 10726366640, 10857001590). As described by one participant, “The weather is tough, very cold weather. Over there [Dubai] there is only one weather, here there's many (011805-24)”. Participants self-managed this transition by recognizing that cold weather is a part of Canadian life (011805-15, 011805-12). One participant explained, “I can’t change the weather according to my likes and dislikes, can I? I have to live with it and it is up to myself how I can make the most of that, right? (011805-15)”. Due to this, participants found alternate exercises such as walking indoors or on a treadmill (011805-15, 10726366640).

### Personal development transition

To conclude their midlife experiences as immigrants, participants primarily expressed feelings of maturity, confidence, independence, and freedom (011805-12, 080101-21, 011805-21). Many participants felt confident due to their life experiences of successfully coping with menopause and migrating to Canada (011805-24, 011805-34, 10857001590, 011805-15). A participant described, “I think I have been through this phase like a star, shining brightly. I had my times when I wasn’t able to manage, maybe, but I still made it through. You know, like stars are always there but you can’t see them because of the sunlight, but they are there all the time, right? And they get their own time to shine when it's night, so I’m like that star (011805-15)”. Participants also expressed feelings of independence and freedom (10726366640, 011805-25, 011805-01, 080101-21). One participant stated, “I’m a free bird. […] I have the knowledge that I think, if I need to survive, I can survive on my own (080101-21)”.

## Discussion

### Findings summary

South Asian immigrant women experienced many different transitions in their midlife experience in Canada. These transitions include changes in their (a) lifestyle, (b) career, (c) family, (d) physical health, (e) mental health, (f) social, (g) environment, and (h) personal development. Women actively managed their transitions using strategies such as exercise, socialization, counselling, and religion. Women expressed the need of social, community, and governmental support to facilitate their midlife transitions.

### Types and patterns of transitions

South Asian immigrant women experience various types of transitions in their midlife, including developmental, situational, and health transitions (Schumacher & Meleis, 1994). The developmental transition includes menopause, aging, changes in family dynamics, social life changes, and personal growth. The situational transition includes transitions related to immigration, such as lifestyle, career, family, social life, personal, and environmental changes. The health/illness transition includes both physical and mental health changes. Our study found that some transitions fell into multiple types because they influenced many parts of the midlife immigrant women's experience. For instance, the lifestyle transition was affected by aspects of menopause, acculturation, and unemployment. These types of transitions occurred simultaneously with other stressors to produce an overall complicated midlife experience. In our study, midlife women experienced multiple and complex transitions as they migrated to a new country, experienced menopausal symptoms, learned a new language, searched for employment, and more.

Our study suggested that participants prioritized their lifestyle and career transitions over their physical health transition. It is common for women to prioritize transitions related to immigration over transitions related to menopause and their health (Mahadeen et al., 2008). Immigrants and refugees face significant financial pressures due to unemployment and may occupy low-level, dead-end jobs due to limited English skills (Yakushko et al., 2008). Our study found that women were compelled to learn English and other new skills to increase their qualifications and be integrated into Canadian society. These demands enabled them to become more independent and achieve personal growth and development. Due to the significant progress they made in their lifestyle and career transitions, women valued their personal development transition and considered personal growth to be their most important midlife achievement. Notably, women may ignore their health care needs because of their busy schedules, thereby necessitating future community services to facilitate women's health care.

### Properties of transition experiences: awareness and engagement

This study found that South Asian women had low awareness and moderate engagement with regards to health transitions. Awareness refers to whether an individual has knowledge about their transition experience. Engagement describes how involved they are in the transition process through seeking information and active preparation (Marnocha et al., 2011; Meleis et al., 2000b). Participants often spent months or years enduring their symptoms before seeking help, and sometimes had trouble finding health educational resources. A study conducted among Ghanaian-Canadian women found that they also had no knowledge about menopause and only started inquiring about it after noticing changes in their bodies (Ohemeng, 2009). Similarly, Swedish women were described as somewhat aware of the physical and psychological changes of menopause, but unaware of how to reduce symptoms and prevent problems (Berterö, 2003).

Participants in our study expressed a strong desire to learn more and improve their understanding of midlife transition but did not know how. While European-American women learned from text and online literature (Marnocha et al., 2011), it has been reported that immigrant women may be more comfortable learning in peer cluster groups. When educating immigrant women, healthcare providers should assess the individual's current knowledge level, desire for treatment, and language comprehension (Hall et al., 2007). Health literacy is a key component of ensuring a healthy transition into the midlife; therefore, it is imperative for healthcare providers and policymakers to create accessible resources for women to learn more about their health transitions.

Contrastingly, in terms of the transitions related to migration, such as lifestyle and career, our study found that participants were highly aware and engaged. Many women took the initiative to utilize community resources such as language centers and city programming, however, they were not completely satisfied with their experiences. They expressed difficulties in finding appropriate employment that matched their education and skillset. Although immigrant women are generally better educated than their Canadian-born counterparts, they are less likely to be employed in positions that correspond to their education level (Chard et al., 2000). Participants had several suggestions for improving the accessibility of such professional supports and resources to facilitate their lifestyle and career transitions. Interventions include individual career counseling to help with job-searches and interviewing strategies, as well as offering structured group workshops in community organizations (Yakushko, 2006). Midlife women may have a smoother career and lifestyle transition if more relevant, specialized, and accessible career services are implemented and advertised in their communities.

### Patterns of response

In our study, South Asian women coped with their challenges with midlife transitions through various self-management strategies. These strategies were regarded as the patterns of response used to identify healthy transition processes (Meleis et al., 2000b). One of the main coping mechanisms used was social connectedness. Although participants stayed in touch with their friends, family, and professionals, they reported that familial support (or lack thereof) greatly influenced their midlife transition. In particular, spousal support was necessary to facilitate a healthy transition, and having good family dynamic allowed participants to advance in other aspects of their midlife transition. This finding is consistent with the literature. A study among immigrant mothers in Quebec, Canada found that compared to non-immigrant mothers, those who received weak spousal support had higher rates of depression (Mechakra-Tahiri et al., 2007). Although the resettlement process can result in a loss of social support, it is essential for midlife women to have supportive social relationships to maintain their functional health and cognitive skills (Lachman et al., 2015). The strong collectivist attitudes and behaviors of Eastern cultures make Asian-Americans more likely to rely on familial relationships for emotional support compared to their Western counterparts (Zhang & Ta, 2009). However, if familial support is limited or non-existent, immigrant women should consider seeking peer or professional support through community services in order to engage in a healthy midlife transition.

Another self-management strategy used by participants in our study was the use of a positive mindset. They expressed that having a positive attitude helped them adapt to new circumstances and face their challenges. Positive beliefs, such as having a sense of control, are protective factors that help individuals in their midlife (Kremen et al., 2012). In the Korean culture, menopause is generally regarded as shameful, and this can lead women to suppress their emotional states, thus negatively impacting their transition outcome (Meleis et al., 2000b). A previous study on South Asian and Latina immigrant women to Canada and Australia identified that negative constructions of menopause were evident, although resistance towards such negative constructions was also notable ( Ussher et al., 2019). Contrastingly, most participants in our study had positive views about the midlife transition and considered it to be a new stage of life. Women who maintained a healthy self-esteem and incorporated positive lifestyle changes experienced more favorable transitions.

Although most of the women in our study had an overall positive personal development transition, some participants held negative views about their physical appearance. There is evidence to suggest that a negative attitude towards menopause is associated with higher body dissatisfaction, appearance-related anxiety, and lower perceived attractiveness (Pearce et al., 2014). However, attitudes about menopause can be improved through psychoeducational programs that improve health literacy and coping abilities. Access to culturally appropriate resources is necessary for immigrant women to learn about health transitions and adopt a positive attitude to cope with the midlife challenges they face.

### Healthcare therapeutics

Therapeutics refer to healthcare provider interventions that can prevent unhealthy transitions, promote well-being, and help women manage their transition experiences (Meleis et al., 2000b). Our study's findings identified three major areas for therapeutic intervention: community and social support for lifestyle and career transitions, community and social support for physical and mental health transitions, and promotion of family-centered care. The following recommendations have been proposed by considering the firsthand experiences of participants in our study.

Immigrant South Asian women suggested that most of the stress they faced revolves around finances and the inability to find employment without going through the upgrade or accreditation process. To ease the lifestyle and career transitions of immigrant women, formal and informal sources of support can be used to build the necessary social networks and facilitate the search for employment (George & Chaze, 2009; Sword et al., 2006). In terms of lifestyle transition, our study found that most participants were unaware of settlement services; however, those that did use them were highly satisfied with their experience. There are many settlement support services in the Greater Toronto Area; however, these agencies have failed to reach their target populations as the data show that a very small percentage of immigrants use them (Islam & Mayer, 2014). These agencies may be able to expand their reach through targeted advertising and improving awareness in immigrant communities. Further research can be conducted in this area to identify the exact reasons for underutilization of settlement services.

In terms of the career transition, career counsellors who provide women with resources for obtaining a job, including job-search skills, access to occupational information, as well as federal, provincial, and municipal laws, rules, and regulations can be employed. Individual counselling may be intimidating for many women, therefore, it may be more suitable to offer structured group workshops and sessions through local community organizations (Yakushko, 2006). Chronister (2006) developed a career intervention program for Mexican immigrant women that was provided through a domestic violence shelter. Moreover, Shea et al. (2007) implemented a culturally-specific career exploration group for Chinese immigrant youth to address their career concerns. These design frameworks can be used as a template when creating a culturally sensitive career intervention for South Asian immigrant women in the Greater Toronto Area. Due to the financial instability of many newcomer families, these services can be subsidized by the government to allow migrant women to obtain good jobs and contribute to the growth of the economy. Moreover, our study showed that midlife women require more accommodations in the workplace, such as additional sick days, to facilitate their physical health transition.

Participants in our study frequently ignored health-related issues due to a lack of awareness and resources. They did not seek help from family or friends due to taboo and secrecy surrounding menopause. This may have exacerbated their negative expectations about the process of menopause (Ussher et al., 2019). Some participants in our study had positive experiences with their family physicians, while others did not find them helpful. To improve women's experience, physicians and healthcare providers should be provided training in cultural competency to better understand the unique barriers of middle-aged South Asian immigrant women, given they are a factor influencing menopausal transition in immigrant women (Zou et al., 2021a; Zou et al., 2021b). These discussions should include the provision of information that will ease the midlife transition, focusing on menopause as a wellness experience that can be self-managed (Mahadeen et al., 2008). Healthcare providers should also begin educating women about menopause before the onset of symptoms since early education can decrease anxiety about the midlife (Marnocha et al., 2011). Moreover, since English is not the first language of many immigrants, culturally-competent and linguistically-appropriate infographics, pamphlets, and websites can be created to educate women about their physical health transition (Stewart et al., 2006).

Helping women receive appropriate mental health support, through support groups and counsellors, may also decrease distress with menopausal symptoms (Hall et al., 2007). It has been reported that South Asian groups hesitate to seek professional help because they have insufficient access to culturally appropriate services or due to the stigma associated with seeking help for mental health issues (Guruge et al., 2015; Samuel, 2009; Stewart et al., 2006). Special efforts must be made from community-organizations to publicize the advantages of professional counselling and increase outreach initiatives to bridge the communication gap with immigrant women (Neufeld et al., 2002; Samuel, 2009). Moreover, our study found that participants expressed a strong desire to connect with like-minded individuals in their community who can sympathize with their experiences. Support groups may be helpful in increasing self-awareness, managing menopausal symptoms appropriately through self-care and healthcare interventions, and connecting with other midlife women (Ahmad et al., 2005; Mahadeen et al., 2008; Stewart et al., 2006). Participants in our study were unaware if such services existed; therefore more attention should be placed on the implementation and promotion of social interventions and peer support groups. Overall, Canada must improve its social and structural support systems to facilitate the successful transition, settlement, and integration of immigrants (Tang et al., 2007). Policies and programs must be made available to provide immigrant women with timely informational, financial, emotional, and culturally-safe support (Guruge et al., 2015).

### Limitations

Selection bias existed since the participants in this study were primarily from India, Pakistan, and Sri Lanka. Although the non-profit agencies and community centers facilitated participant recruitment, it was difficult to acquire participants from other South Asian countries such as Bangladesh, Bhutan, Nepal, Maldives, and Afghanistan. However, it is important to note that the diversity of the study sample is reflective of the current immigrant population in Canada where the majority of South Asian immigrants are from India and Pakistan (Statistics Canada, 2017b). Moreover, the qualitative nature of the study precludes the possibility of drawing a causal relationship between the potential interventions and midlife transition of these women.

### Implications to practice and policy

The findings from this study can help guide future interventions and policy changes for South Asian immigrant women in Canada. First, governments need to create better employment policies to facilitate immigrant women settlement, transferral of skills, and re-employment in Canada. These can include changes at the government level with foreign credential recognition or community-level changes through career counselling and networking opportunities. Moreover, health care and community services to promote physical and mental health should be emphasized. Cultural competency training should be provided to healthcare professionals, in order for them to provide their immigrant patients with resources and information on the physical challenges associated with aging and menopause. Importantly, mental health supports should be also considered as a priority for this group in order to help them manage their symptoms and connect with other midlife women.

### Recommendations for future studies

This study provides valuable insight into the midlife experiences of South Asian immigrant women by elucidating eight transitions experienced by this population, something previous literature had not yet explored Further research is needed to design an appropriate intervention to aid South Asian immigrant women in the transition processes identified in this study, and evaluate its acceptance and effectiveness. Various types of supports have been recommended by participants; therefore, a study assessing the implementation of a specific intervention for midlife women is warranted. Future studies should also evaluate the impact of the COVID-19 pandemic on the midlife transition for this population. Technology-based interventions should be explored as a potential avenue of support as they can be easily incorporated into busy lifestyles and serve as a safe alternative for in-person activities.

## Conclusion

This qualitative study investigated the midlife experiences of twenty-two South Asian immigrant women in the Greater Toronto Area.. Qualitative content analysis elicited eight transitions including (a) lifestyle, (b) career, (c) family, (d) physical health, (e) mental health, (f) social, (g) environment, and (h) personal development. Women actively managed their transitions using strategies such as exercise, socialization, counselling, and religion. However, women expressed the need for social, community, and governmental support to facilitate midlife transitions. Implications for future improvement include implementingtherapeutic interventions, specifically,community and social support for lifestyle and career transitions, community and social support for physical and mental health transitions, and promotion of family-centered care. To promote healthy midlife transition, governments need to create better employment policies to facilitate immigrant women settlement, transferring skills, and re-employment in Canada. In addition, health care and community services to promote physical and mental health among this group should be emphasized.
